# Socio-Economic Inequalities in Access to Drinking Water among Inhabitants of Informal Settlements in South Africa

**DOI:** 10.3390/ijerph181910528

**Published:** 2021-10-07

**Authors:** Marieke J. Oskam, Milena Pavlova, Charles Hongoro, Wim Groot

**Affiliations:** 1 Department of Health Services Research, CAPHRI, Maastricht University Medical Centre, Faculty of Health, Medicine and Life Sciences, Maastricht University, PO Box 616 6200MD Maastricht, The Netherlands; m.pavlova@maastrichtuniversity.nl (M.P.); w.groot@maastrichtuniversity.nl (W.G.); 2 Peace and Sustainable Security (PaSS), Developmental, Capable and Ethical State Division, Human Sciences Research Council, 134 Pretorius Street, Private Bag X41, Pretoria 0001, South Africa; CHongoro@hsrc.ac.za; 3 School of Health Systems and Public Health, University of Pretoria, Private Bag X323, Pretoria 0001, South Africa

**Keywords:** socio-economic inequalities, access, drinking water, informal settlements, South Africa

## Abstract

While evidence from several developing countries suggests the existence of socio-economic inequalities in the access to safe drinking water, a limited number of studies have been conducted on this topic in informal settlements. This study assessed socio-economic inequalities in the use of drinking water among inhabitants of informal settlements in South Africa. The study used data from “The baseline study for future impact evaluation for informal settlements targeted for upgrading in South Africa.” Households eligible for participation were living in informal settlements targeted for upgrading in all nine provinces of South Africa. Socio-economic inequalities were assessed by means of multinomial logistic regression analyses, concentration indices, and concentration curves. The results showed that the use of a piped tap on the property was disproportionately concentrated among households with higher socio-economic status (concentration index: +0.17), while households with lower socio-economic status were often limited to the use of other inferior (less safe or distant) sources of drinking water (concentration index for nearby public tap: −0.21; distant public tap: −0.17; no-tap water: −0.33). The use of inferior types of drinking water was significantly associated with the age, the marital status, the education status, and the employment status of the household head. Our results demonstrate that reducing these inequalities requires installing new tap water points in informal settlements to assure a more equitable distribution of water points among households. Besides, it is recommended to invest in educational interventions aimed at creating awareness about the potential health risks associated with using unsafe drinking water.

## 1. Introduction

Safe drinking water is essential for human health and can prevent the development of illnesses such as diarrheal disease and enteropathy, which could lead to death, especially in children below the age of five [1,2,3,4,5,6,7,8]. The World Health Organization (WHO) defines safe drinking water coverage as the proportion of the population with access to an adequate amount of safe drinking water located within a convenient distance from the user’s dwelling [9]. In 1990, one out of four people in the world did not have access to safe drinking water sources [9]. The situation has improved over the years as a result of policy interventions guided by the United Nations Millennium Development Goals (MDG), which urged to halve the proportion of people without sustainable access to safe drinking water by 2015 [8,9,10,11]. Many countries have achieved universal access to safe drinking water in response to these goals. Indeed, in 2015, 91% of the global population used a safe drinking water source, but still 663 million people lacked access [9]. These people are forced to collect water from unprotected and often contaminated wells or springs, or they have to use surface water and store the water in containers until use, resulting in poor health outcomes [9]. The problem of access to drinking water is especially prominent in Sub-Saharan Africa and South Asia, where less than 75% of the population has access to safe water facilities [9,12,13,14]. This rate is even lower in rural areas and among disadvantaged socio-economic groups [9,15,16,17,18]. Although disparities in access to safe drinking water vary across the countries, in all contexts, they present a barrier to achieving universal safe-water access [10,19].

The lack of access to safe drinking water is a human right problem. Hence, the new Sustainable Development Goals (SDG) include the objective of achieving access to safe and affordable drinking water for all by 2030 [20]. The drinking water problem also has a considerable economic impact. Specifically, unsafe or distant water sources result in a loss of economic productivity and foregone benefits due to time spent collecting water [21]. In addition, the transport, handling, and storage of drinking water increases the risk of contamination and can lead to poor health outcomes [22,23,24,25]. It has been estimated that the health consequences of the lack of access to safe drinking water and poor sanitation together can account for up to 7% of the gross domestic product, not even including social and environmental consequences [9,20]. The lack of access to safe water sources is also linked to social issues, such as less school attendance, especially among girls who are menstruating [26]. Greater access to safe water could thus lead to substantial increases in time available for work, attending education, and raising families and can improve health outcomes by preventing the use of contaminated water [27]. Therefore, the topic has been a key research theme around the world.

Previous research has primarily focused on investigating the access to drinking water at a national level or has disentangled the urban–rural differences [10]. The availability of new disaggregated data on the access to drinking water among other key population groups would enable the analysis of access to safe drinking water by marginalized groups like slum populations [20]. In this study, we focused on the access to drinking water among those living in informal settlements, which has barely been the focus of previous studies [28]. We investigated the case of informal settlements in South Africa specifically, which has not been adequately done before. Therefore, our study is of interest to researchers, water practitioners, and decision-makers in South Africa as well as other countries dealing with water-related public health problems in informal settlements.

In particular, this study aimed at assessing socio-economic inequalities in access to drinking water among inhabitants of informal settlements in South Africa. Access to drinking water among less advantaged groups was compared to the access among more advantaged groups in those settlements. The study provides a base for outlining implications for public health policy. Further in this section, we present the study context as well as the key concepts applied in our investigation.

### 1.1. Study Context

About 65% of South Africa’s population lives in urban areas [9]. One out of four inhabitants of the urban population, approximately 8 million people, live in informal settlements [29]. There are informal settlements in the rural areas as well. Informal settlements are housing areas that are often illegally built on municipal land, and often these areas lack access to basic services, such as sewerage, electricity, roads, and clean drinking water [30]. Many informal settlements in South Africa originate from the apartheid era (1948–1994), in which people were systematically segregated by national policies based on race, and this affected their housing, job opportunities, and education. As a result, many non-white inhabitants moved to informal settlements in rural areas or areas surrounding the cities. While the apartheid was dismantled in 1994, gaps between wealth and extreme poverty still exist in South Africa, which is the reason why informal settlements continue to exist today [30].

The problem of access to safe drinking water in those settlements has been acknowledged. While South Africa met the 2015 MDG target, with over 91% of the population having access to safe drinking water sources at that point, there is still a group of over 3.5 million people who lack sustainable access to safe drinking water [9]. This group mainly consists of inhabitants of informal settlements located in rural areas. Specifically, in rural areas, 38% of the people use piped tap water on premises, 43% use other safe drinking water sources such as protected wells and springs, 12% use unprotected sources of water, and 7% use surface water [9]. The urban population is much better off, with virtually all urban residents now having access to safe drinking water sources: 92% of the urban population uses piped water on premises, and 8% uses other improved sources of drinking water [9]. Nevertheless, also in urban settings, there is still room for improvement as 28 million people, mainly inhabitants of informal settlements, do not yet have piped water on premises [9]. While evidence from a number of developing countries suggests the existence of socio-economic inequalities in access to safe drinking water [31,32,33], studies on this topic have rarely focused on informal settlements and especially not in South Africa.

### 1.2. Access to Safe Drinking Water

In this study, two aspects of access to drinking water were considered: the safety and the proximity of the main water source used by a household. These aspects are presented below through definitions that have been used in previous studies as well.

With the aim to classify the safety of drinking water around the world, the WHO provides the “drinking water ladder,” which includes four levels, namely, from the most- to the least-safe drinking water level [9]:

▪ Piped water on premises: when there is a piped household water connection located inside the user’s dwelling, plot, or yard.
▪ Other improved drinking water sources: public taps or standpipes, tube wells or boreholes, protected dug wells, protected springs, and rainwater collection.
▪ Unimproved drinking water sources: unprotected dug wells, unprotected springs, carts with small tanks/drums, tanker trucks, and bottled water.
▪ Surface drinking water sources: water from rivers, dams, lakes, ponds, streams, canals, and irrigation channels.

In addition, the World-Wide Fund for Nature provides a country-specific ranking of the safety of drinking water in South Africa [34]. The spectrum of water safety is ranked from the most- to the least-safe based on five levels:

▪ Piped water inside the dwelling.
▪ Piped water inside the yard.
▪ Piped water on a community stands at a distance smaller than 200 m from dwelling.
▪ Piped water on a community stands at a distance greater than 200 m from dwelling.
▪ No access to piped water.

The threshold of 200 m is used because it is the South African target for basic services. An important difference between this classification and the WHO classification is that here the source proximity is also incorporated.

In the South African Guidelines for Human Settlement Planning and Design, a distinction has been made based on the water source proximity [35]:

▪ Nearby: water source at a distance less than 250 m from dwelling.
▪ Medium distance: water source at a distance of 250–1000 m from dwelling.
▪ Considerable distance: source at a distance greater than 1000 m from dwelling.

**Figure 1:**
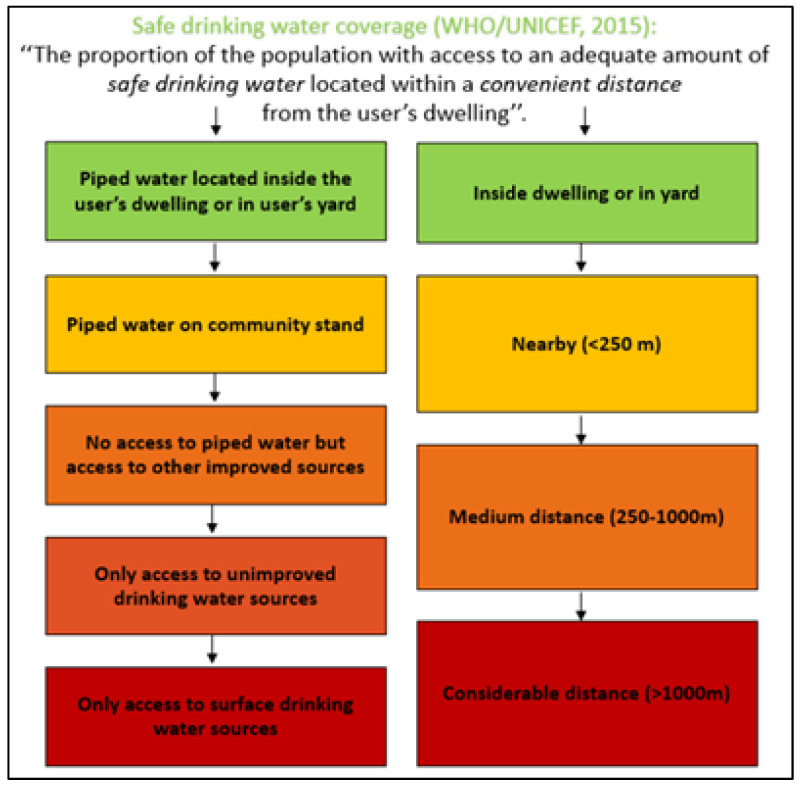
Safety and proximity of drinking water sources [9,34,35].

In Figure 1, the above definitions are combined to illustrate the safety and proximity aspects of drinking water sources. In our study, we applied this model to define access to drinking water in informal settlements in South Africa. In the next section, we provide further details on how drinking water use was operationalized and measured.

### 1.3. Socio-Economic Inequalities

One of the principles in the Constitution of the WHO from 1946 states: “The enjoyment of the highest attainable standard of health is one of the fundamental rights of every human being without distinction of race, religion, political belief, economic or social condition” [36]. This principle is closely related to the ethical value of equity in health. In 1992, Whitehead described health inequities as differences in health that are unnecessary, avoidable, unfair, and unjust [37]. The article of Whitehead succeeded in raising awareness and stimulating debate on the ethical value of equity in health [38]. Equity in health means that all social groups have an equal opportunity to be healthy, which requires that resources and programs are distributed in ways that most likely equalize the health outcomes of disadvantaged social groups with the health outcomes of more advantaged social groups [38].

An important social determinant of health is access to safe drinking water [1,2,3,4,5,6,7]. Inequalities in safe drinking water put population groups who are already socially disadvantaged (for instance, by virtue of being poor, female, or member of a particular racial, ethnic, or religious group) at a further disadvantage [8,9,10,11,12,13]. Assessing inequalities in access to safe drinking water requires a comparison of more and less advantaged social groups with regard to access to drinking water. Knowledge on socio-economic inequalities in access to safe drinking water is a starting point for (re)formulating public health policies targeting the provision of adequate water-supply systems to people who do not yet have sustainable access. Our study provides evidence on the inequalities in access to safe drinking water among inhabitants of informal settlements in South Africa.

For our investigation, we used concentration indices and concentration curves to describe inequalities. This method has long been used to measure inequalities [39]. The concentration curve illustrates how a specific feature (in this study, the drinking water source used) varies across the distribution of wealth. The concentration curve is compared to the line of perfect equality. When there is no considerable difference between the most and least wealthy, the concentration curve is close to or coincides with the equality line, which indicates the absence of inequalities. If the use of a given drinking water source is more prevalent (more concentrated) among the wealthier individuals, the concentration curve for that water source will be considerably below the equality line, and if the use is more prevalent among the less wealthy individuals, the concentration curve will be considerably above the equality line. We can measure the degree of concentration through the concentration index. In other words, the concentration index shows how the concentration curve deviates from the equality line (magnitude and direction). Thus, the concentration index reflects the scale of the social gradient. In this study, we estimated concentration indices for different population subgroups for different types of water sources. The following section provides further details on the estimation and interpretation of the concentration indices and concentration curves in this study.

We expect that wealth and other socio-demographic characteristics, like those identified in previous studies [8,9,10,11,12,13] and in particular for South Africa [40], are associated with inequalities in access to safe drinking water in informal settlements as well.

## 2. Materials and Methods

To investigate socio-economic inequalities in access to drinking water among inhabitants of informal settlements in South Africa, this study applied a positivist research paradigm, which implies that existing theory is used to develop hypotheses (expectations) that are examined during the research process. Our study thus had an explanatory nature. Explanatory studies aim to better understand a given study phenomenon and can provide insight into the association between that phenomenon and a set of explanatory factors. In our study, we explained inequalities in access to drinking water among inhabitants of informal settlements in South Africa (our study phenomenon) by providing evidence on the association between that phenomenon and socio-economic characteristics.

We considered those inhabitants’ socio-economic characteristics that have been reported to be relevant determinants of access to drinking water in previous studies [8,9,10,11,12,13,40]. Thus, we followed a deductive line of reasoning to formulate our prior expectation, as stated above. In particular, we expected to find the same associations between inequalities in access to safe drinking and socio-economic characteristics as those reported in previous studies. To frame our study, we divided these characteristics into three categories:

▪ Economic factors. This group includes factors, such as household wealth, which determine the affordability of safe water sources within or close to the dwelling. As reported in the literature, poor households are more likely to use unsafe water sources and/or distant water sources than wealthy households [31]. This discrepancy refers to wealth-related inequalities.
▪ Demographic factors. In this group, gender, age, and household size were included since these factors could also cause disparities in access to drinking water. For example, when water is not available at the dwelling, women are most often responsible for fetching and storing water for the household [13]. This implies gender inequalities since women can spend less time on other social activities. There could also be intergenerational inequalities since older heads of households are found to be more ready to pay for water than younger heads [40].
▪ Social factors. This group includes education, marital status, employment, ethnicity, and other factors, which are reported to be critical dimensions of the use of drinking water and which are related to social inequalities in access to safe drinking water [13,40]. Individuals with higher education might be more aware of the importance of good quality water than those with lower education. Those with employment might have more resources to ensure water at the dwelling, while the unemployed might have more time to do so.

These three groups of factors may have a different association with access to drinking water depending on the specific context [16,17]. Thus, context-related variations can also cause inequalities [10]. However, we focused here on one specific context, namely, informal settlements in South Africa (see Section 1.1).

The type of our study was quantitative, which means that we use quantitative data and statistical methods to investigate socio-economic inequalities in access to drinking water among inhabitants of informal settlements in South Africa. We used data collected at a single point in time, which made our study design cross-sectional. Details about the data collection and the statistical techniques used in the analyses are presented below.

### 2.1. Data Collection

This study used data from “The baseline study for future impact evaluation for informal settlements targeted for upgrading in South Africa,” a cross-sectional study conducted by the Human Sciences Research Council (HSRC) in Pretoria between July and September 2015. As part of this study, a household survey was conducted using a structured questionnaire consisting of a large number of questions divided over twelve domains, including: [1] household roster; [2] education; [3] economic activity; [4] health, food, and nutrition security; [5] borrowing, credit, and savings; [6] microenterprise; [7] housing and tenure; [8] infrastructure and service delivery; [9] satisfaction; [10] social capital, social network, and community participation; [11] crime and safety; and [12] attitude towards foreigners.

Households eligible for participation were living in informal settlements targeted for upgrading (i.e., upgrading of the settlement was included in the medium-term municipal budgets). To select households, first, informal settlements were selected from each province by stratified random sampling (i.e., informal settlements were stratified by province, and a random sample of informal settlements was selected for participation in each stratum). Thereafter, from each informal settlement included in the study, a random sample of 45 households was selected for participation in the study. Prior to the baseline study, a pilot study was conducted in three informal settlements in Gauteng, after which the household survey and logistics underwent some adaptations.

The household survey tools were approved by South Africa’s Department of Human Settlement (DHS) and the Department of Performance Monitoring and Evaluation (DPME) and were used in the training of fieldworkers. In total, data were collected from 3202 households living in 78 informal settlements across all provinces of South Africa. The research protocol for “The baseline study for future impact evaluation for informal settlements targeted for upgrading in South Africa” was approved by the Research Ethics Committee of the HSRC, and the use of this data for the purpose of our study was approved by Maastricht University. Written informed consent was obtained from all participants, and information that revealed their identity was treated as confidential.

### 2.2. Dependent Variable: Drinking Water Use

Based on the safety and proximity aspects of drinking water sources presented in Figure 1 and given the data available in our dataset, we defined four categories of drinking water use. These four categories were distinguished based on both the type of drinking water source and the distance from the user’s dwelling to the drinking water source. Ranked from best to worst, those categories were: (1) piped tap water in the dwelling or on-site (hereafter referred to as “piped tap on property”), (2) public tap water at a distance less than 200 m from the dwelling (hereafter referred to as “nearby public tap”), (3) public tap water at a distance of 200 m or more from the dwelling (hereafter referred to as “distant public tap”), and (4) another drinking water source, which is not tap water, at any distance from the dwelling (hereafter referred to as “no-tap water”).

### 2.3. Explanatory Variables: Household Characteristics

As explained above, we considered the inclusion of three groups of explanatory variables based on previous studies, namely, economic, demographic, and social characteristics. Given the available data, the following demographic characteristics were used as explanatory variables in the analyses: household size (<4 members/≥4 members), gender of the household head (male/female), and age of the household head (<35 years/35–50 years/>50 years).

In addition, we included a set of social characteristics available in the dataset, such as nationality of the household head (South African/other), marital status of the household head (partner/no partner), education status of the household head (low: no schooling, reception phase and foundation phase, i.e., grade R-3/intermediate: intermediate phase and senior phase, i.e., grade 4–9/high: further training and education phase, i.e., grade 10–12 and higher education), and employment status of the household head (employed/unemployed).

Finally, we included one economic characteristic, namely, the socio-economic status (SES) as indicated by a wealth index. It was calculated based on a principal component analysis (PCA) on housing characteristics, access to basic services, and ownership of durable assets. In order to create the wealth index, all nominated variables were re-coded into binary variables, and in case one of the two categories contained less than 5% of the households, that variable was excluded from the PCA model based on the assumption that that variable was not able to distinguish between higher and lower SES. For the same reasons, variables were excluded in the case of intervariable correlations lower than 0.1 or higher than 0.9. Unlike other wealth indices, the variable drinking water use was not included to avoid endogeneity in the analyses due to the existence of a correlation between explanatory and dependent variables. In the initial PCA, the following variables were included: electricity, fridge, deep freezer, microwave, electric gas stove, washing machine, iron, TV set, VCR/DVD player, M-Net and/or DStv subscription, Hi-Fi music center, fan, and motor vehicle. By means of the PCA, the data were reduced to a set of orthogonal principal components, from which the first component, which explained most of the variation, was used as the wealth index. Subsequently, the first component was transformed into a categorical variable with five categories or quintiles, indicating the SES of households. The first quintile contained the households with the lowest SES and the fifth quintile the households with the highest SES. A second PCA was performed excluding the variables fan and motor vehicle ownership, as these variables showed an illogical distribution of the outcome over the SES quintiles.

### 2.4. Statistical Analyses

All analyses were performed using the software package SPSS version 24. In total, 1913 households (59.7%) had a missing value on at least one essential variable, and therefore, it was deemed necessary to impute missing values. However, 918 households (28.7%) had missing values on more than three of the essential variables, and as this does not provide a good basis for missing data imputation, it was decided to exclude those households from the analyses, resulting in a final study population of 2284 households. To impute the remaining part of the missing data, we used multiple data imputations by chained equations. Specifically, information on other variables in the dataset was used to predict and impute the missing values. Logistic regression was used as the imputation method. Each missing value was imputed ten times, and Rubin’s rules were used to combine the results [41].

To investigate which household characteristics were associated with drinking water use, multinomial logistic regression analyses were performed. First, univariate multinomial logistic regression analyses were conducted for each explanatory variable separately. In case an explanatory variable showed a *p*-value ≤ 0.10 in the univariate analysis, it was included in the multivariate model, for which *p*-values ≤ 0.05 were considered statistically significant. Prior to the multivariate multinomial logistic regression analysis, we checked for the presence of multicollinearity between explanatory variables.

Besides the use of regression analyses, inequalities in drinking water use were also estimated and visualized by means of concentration indices and concentration curves. As mentioned earlier, the concentration curve and index were used as measures of inequality across social groups. The interpretation of these two measures is as follows. If the concentration index is zero, there is no substantial inequity; if it is positive, then we have a concentration among the wealthier individuals; and if it is negative, then we have a concentration among the less wealthy individuals. Concentration indices are visualized by means of concentration curves, in which the *X*-axis indicates the cumulative percentage of the study population ranked by SES (beginning with the lowest), and the *Y*-axis indicates the cumulative percentage of the characteristic being studied corresponding to each cumulative percentage of the distribution of the SES indicator. When the curve lies above the line of equality, the concentration index has a negative value, which implies that the use of that particular type of drinking water is disproportionately concentrated among people with lower SES, and when the curve lies below the line of equality, it is the other way around. Concentration indices and concentration curves were calculated in this study in Microsoft Excel version 1808 based on the method described by Kakwani, Wagstaff, and Doorslaer [42], and they were calculated for the total group and for different population subgroups.

## 3. Results

**Figure 2:**
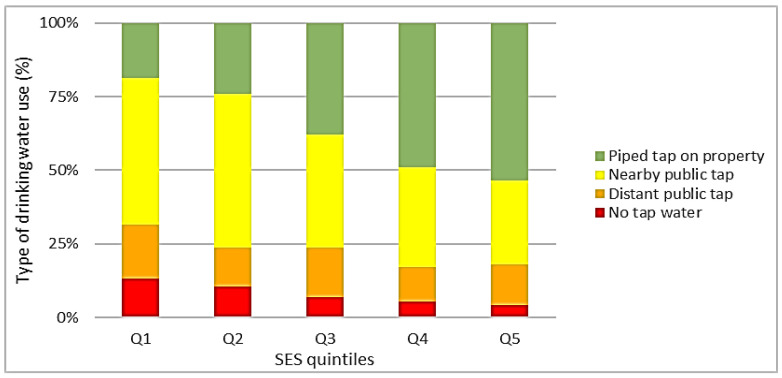
Type of drinking water use stratified by socio-economic status (SES) quintiles, in which the first quintile (Q1) includes the households with the lowest SES and the fifth quintile (Q5) the households with the highest SES.

The economic, demographic, and social characteristics of the total group of 2284 households and the distribution of type of drinking water use over the total group and over different population subgroups are displayed in Table 1 and Figure 2, respectively.

As shown in Table 1, of all households, 36.0% had a piped tap on their property, 40.5% used a nearby public tap, 15.3% used a distant public tap, and 8.3% did not have access to tap water at all. In total, 49.9% of the households consisted of four or more household members. From the household heads, 52.1% were male, 95.9% had a South African nationality, 48.1% had a partner, and 38.9% were employed. Furthermore, 31.9% of the household heads were below the age of 35, 39.0% were aged between 35 and 50 years, and 29.1% were older than 50 years. Moreover, 18.4% of the household heads had a low level of education, 39.9% an intermediate level, and 41.7% were highly educated.

**Figure 3:**
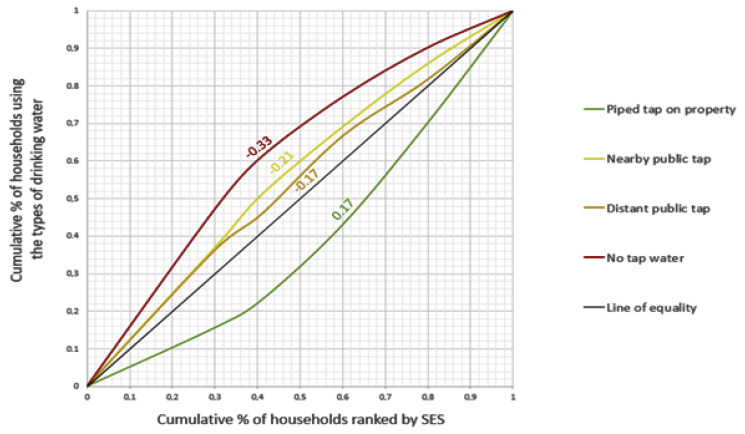
Socio-economic inequalities in the use of drinking water as represented by concentration curves, with corresponding concentration indices displayed alongside the graphs; SES = socio-economic status.

Figure 2 illustrates the type of drinking water use by SES quintiles. Besides, Figure 3 illustrates the related socio-economic inequalities (concentration curves). As shown in these figures, the use of a piped tap on property ranged from 18.7% in the first SES quintile, corresponding to the lowest SES, to 53.5% in the fifth quintile, corresponding to the highest SES (Figure 2). The use of a piped tap on the property was thus disproportionately concentrated among households with higher SES, which was confirmed by a concentration curve below the line of equality and a concentration index of +0.17 (Figure 3). In contrast, the use of a nearby public tap ranged from 52.0% in the second SES quintile to 28.5% in the fifth quintile. The use of a distant public tap ranged from 18.4% in the first quintile to 11.5% in the fourth quintile, and the use of another drinking water source (not tap water) ranged from 13.1% in the first quintile to 4.1% in the fifth quintile (Figure 2). Thus, the use of these inferior (less safe or distant) sources of drinking water was disproportionately concentrated among households with lower SES, which was confirmed by concentration curves lying above the line of equality and concentration indices of −0.21, −0.17, and −0.33, respectively. This indicated that socio-economic inequalities were largest in the group of people not using tap water (Figure 3). In Appendix A, concentration indices are presented separately for different population subgroups.

Table 2 presents the results of the multivariate multinomial logistic regression analyses, which indicate the associations between household characteristics and drinking water use. As all household characteristics showed a significant association (*p* ≤ 0.10) with drinking water use in univariate analyses (Appendix B) and no problems of multicollinearity were encountered, all household characteristics were included in the multivariate model (Table 2).

In comparison with the fifth SES quintile, households in the first quintile had significantly higher odds of using a nearby public tap (odds ratio (95%-CI): 5.12 (3.68, 7.12)), a distant public tap (3.76 (2.53, 5.58)), and of using no-tap water (8.91 (4.88, 16.28)) than of using a piped tap on property. With regard to households in the second and third SES quintile, similar associations were found, which were also statistically significant but less strong (Table 2).

Household size and gender of the household head were not significantly associated with drinking water use. However, the age of the household head was significantly associated with the use of drinking water. In comparison with households having a household head older than 50 years, households having a household head below the age of 35 had significantly higher odds of using a nearby public tap (1.76 (1.29, 2.40)) and of using a distant public tap (1.76 (1.13, 2.75)) than of using a piped tap on property. Furthermore, these households had higher odds of using no-tap water than of using a piped tap on property, but this result was not statistically significant at a significance level of *p* ≤ 0.05 (1.67 (0.99, 2.80)). With regard to households having a household head between 35 and 50 years, similar associations were found, which were also statistically significant but slightly less strong (Table 2).

As shown in Table 2, in comparison with households having a household head with a nationality different from South African nationality, households having a household head with South African nationality had lower odds of using no-tap water than of using a piped tap on property. However, this association was not significant at the *p* ≤ 0.05 level (0.53 (0.25, 1.11)).

Furthermore, in comparison with households with a household head having a partner, households having a household head without a partner had lower odds of using a nearby public tap (0.74 (0.58, 0.93)) and of using a distant public tap (0.76 (0.56, 1.02)) than of using a piped tap on property. However, only the first association was statistically significant at the *p* ≤ 0.05 level.

Additionally, in comparison with households having a highly educated household head, households having a household head with a lower level of education had significantly higher odds of using no-tap water than of using a piped tap on property (2.44 (1.40, 4.25)).

Lastly, in comparison with households having a household head that was employed, households having a household head that was unemployed had significantly lower odds of using a nearby public tap than of using a piped tap on property (0.68 (0.55, 0.85)). In addition, these households had lower odds of using no-tap water, although this association was not statistically significant at the *p* ≤ 0.05 level (0.71 (0.49, 1.02)).

## 4. Discussion

### 4.1. Socio-Economic Inequalities in the Use of Drinking Water

This study is the first study that comprehensively assessed socio-economic inequalities in the use of drinking water among inhabitants of informal settlements in South Africa. The results show that the use of a piped tap on the property was rather low and disproportionately concentrated among households with higher SES (based on the wealth index). Households with lower SES were often limited to the use of inferior (less safe or distant) sources of drinking water. Furthermore, the use of inferior sources of drinking water was significantly associated with the employment status, the age, the marital status, and the education status of the household head. These findings confirm our prior expectation that inequalities in access to safe water sources in informal settlements are associated with economic, demographic, and social characteristics. Thus, the situation in the informal settlements in South Africa, in general, reflects the inequalities observed in previous studies. Although safe drinking water sources are needed for all inhabitants of informal settlements, the most disadvantageous groups should still be the target of immediate policy interventions in those settings. As recommended in other studies [10], community-level interventions could be especially valuable to reach those most in need.

Specifically, as in many previous studies [31,40,43,44,45,46], we found that wealth is an essential factor in access to a safe drinking water source in informal settlements. Although the SES in informal settlements is generally low [28], the use of a piped tap on the property is still disproportionately concentrated among households with higher SES (positive concentration index). To diminish these inequalities and ensure consistent and quality access to drinking water for all, investment in piped drinking water is needed [10]. Such an intervention could be costly, but it could increase the opportunity for labor participation among adults and school attendance among children in informal settlements, who do not need to spend time on securing drinking water from distanced sources [20].

We also found that households with an employed household head more often used one of the inferior (less safe or distant) sources of drinking water than households with an unemployed household head. This finding is counterintuitive but reported in other studies as well [43]. Moreover, in our study, this finding only refers to formal employment at the time of the survey. It should be mentioned that the survey adopted a very short time horizon (i.e., only one week) and did not provide data to identify informal employment. It is well known from previous research that informal employment plays an important role in low- and middle-income countries. The United Nations carried out a study on informal employment in which they concluded that nearly two-thirds of the global workforce participate in the informal economy, and 93% of the world’s informal employment takes place in low- and middle-income countries, including South Africa [47]. Therefore, in our study, employment should not be seen as an indication of being better off since we lack comprehensive employment data. It could be that those in formal employment have less time to secure access to a superior water source, i.e., piped tap on property. This explanation is hypothetical and needs to be a subject of further investigation.

According to our study, households with a younger household head more often used inferior (less safe or distant) sources of drinking water than households with older household heads. This result is in line with a previous study carried out in South Africa, in which factors were identified that explained household payment for potable water [40]. In that previous study, an older age of the household head was associated with the payment for potable water. The authors suggested that this finding might be explained by the prevalence of unemployment among youths in South Africa (46.3% in quarter 1 of 2021, according to Statistics South Africa), which might make payment for water more difficult for younger household heads (in informal settlements) [40]. Our data did not show this kind of relationship between age and employment status, most likely because water is highly subsidized or generally free in informal settlements. It should be mentioned, however, that some studies found no relation between age and use of water sources [45], and some studies did not include this explanatory variable [44].

Households with a household head with a partner more often used one of the inferior types of drinking water (i.e., nearby public tap) than households with a household head without a partner. This finding is in contrast with a study carried out in Indonesia [43]. Specifically, the study conducted in Indonesia reports that households with married or ever-married heads are more likely to use better drinking water sources. At the same time, a study carried out in Ghana found results similar to ours. However, the significant relationship between marital status and access to improved drinking water disappeared after controlling for the place of residence and the region of residence [44]. As explained by the authors, this implied that the association between marital status and water access was fully mediated by those variables [44]. The difference in the findings could also be due to the diversity in the measurement of this explanatory variable. Future studies are needed to provide clarity on the relation between water access, marital status, and place and region of residence. Our results do not offer further insight into these relations.

Households with a household head with a lower level of education more often used another drinking water source instead of piped tap water when compared with households with a household head with a higher level of education. This finding aligns with studies carried out in Indonesia, Vietnam, Ghana, and some other Sub-Saharan African countries. Those studies suggest a positive relationship between the educational attainment of the household head and the probability of having access to improved water sources [43]. The relation between education and access to drinking water sources may be attributed to the fact that household heads with better education are more knowledgeable about the potential health risks associated with using unsafe drinking water [43,44,45,46,48]. Therefore, they make extra efforts for investing in better access to water facilities and securing better quality water [44].

We did not find significant results for household size and the gender of the household head, and the result for the nationality of the household head was not highly significant. This means that we did not find substantial inequalities in the use of drinking water sources among those socio-demographic groups in informal settlements. Since this is the first comprehensive study of its kind for South Africa’s informal settlements, we consider it important that our study is replicated based on other new datasets to establish the validity of our findings. In particular, the absence of statistically significant results for the gender of the household head requires attention since other studies have reported that female household heads are more likely to secure access to better drinking water sources [43,44,45].

### 4.2. Implications for Public Health Policy

Despite the progress made over the last two decades, there is still much room for improvement as only about a third of the households living in South Africa’s informal settlements have access to a piped tap on their property, as shown in our study. The remaining households have access to a nearby or distant public tap or do not have access to piped tap water at all. It should be mentioned that, in South Africa, the majority of the urban poor, particularly those who live in informal settlements, receive a 100% government subsidy, meaning that they do not pay for water. The upgrading of informal settlements remains a policy priority in order to improve access to basic services (water, electricity, and sanitation) among the majority of the poor populations in both urban and rural areas.

The group with no access to piped tap water deserves particular attention and priority in policy interventions due to potential health threats. Households with no access to piped tap water are limited to the use of inferior drinking water sources. However, these water resources might be polluted due to industrial and household waste, making them potentially unsafe for humans [8,23]. Pollution could be the result of broken septic tanks, irrigation, and run-off water containing agrochemicals, which could spread infectious diseases, including cholera and typhoid [28]. Thus, the lack of access to piped tap water might result in poor health outcomes and the spread of diseases due to the use of contaminated water sources [9].

Attention also needs to be paid to households who only have access to a distant public tap. The members of those households spend significant time collecting drinking water, which implies that this time can no longer be spent on work, attending education, and raising families [21,27]. Besides, these households often store drinking water, which increases the risk of contamination and might also result in poor health outcomes [22,23,24,25]. As a result, this group of households also faces health risks related to their drinking water consumption. It is therefore essential to assure the proximity of safe drinking water. Moreover, safe water sources within or near the dwelling offer comfort to household members, especially to women and girls who often have the responsibility to fetch water, which allows them to engage more often in other activities, such as income-generating activities, childcare, and education [20,21,26].

Thus, in order to improve the current situation in South Africa’s informal settlements, short-term governmental interventions are required in order to provide access to piped tap water, even though at a distance, to inhabitants of informal settlements who do not yet have access to piped tap water. Subsequent interventions are needed as well in order to provide access to piped tap water within 200 m from the dwelling of inhabitants of informal settlements who do not yet have access to a nearby public tap. No pretense is made that informal settlements are the only priority. Such interventions should also be applied to other settings in South Africa where access to safe drinking water is problematic, although this has not been the subject of our study.

It should be acknowledged, however, that “improved” water sources might suffer the same problem if the water quality is not appropriately safeguarded. For example, traces of *E. coli* are reported in “improved” water sources as well [8]. Therefore, the improvement of access to safe drinking water sources in informal settlements and other areas should go hand in hand with the proper management of the entire water supply system.

Besides, our results show that the use of piped tap water is associated with the level of education of the household head. In view of this, we would also recommend investing in educational interventions aimed at creating awareness about the potential health risks associated with using unsafe drinking water. Furthermore, as suggested in previous studies [8,19], isolated interventions solely focused on improving access to drinking water sources have questionable success. This is because of the close association between sanitation management and water quality [28]. From a public health point of view, it is crucial to couple the water-improvement interventions with interventions for improving sanitation infrastructure. Such combined interventions are found to have greater health benefits, like increased reduction in diarrheal disease in children under five [19].

These interventions are essential steps to reduce the existing socio-economic inequalities in access to drinking water, which put population groups who are already socially disadvantaged by virtue of being poor at a further disadvantage. There are no robust estimations of the welfare consequences of poor access to safe drinking water, as well as sanitation and hygiene, but these consequences are potentially very large [20]. Further understanding of these consequences and their variation across the socio-economic groups is imperative for effectively prioritizing future policy interventions [31].

### 4.3. Strengths and Limitations

Unlike some other studies conducted in this area, this study combines the type of drinking water source and the distance from the user’s dwelling to the drinking water source, in order to create four categories. Not all previous studies took into account the distance from the user’s dwelling to the drinking water source. However, a long distance from the dwelling to the water source might lead to storage of water, which might in turn lead to consumption of contaminated water and poor health outcomes [22,23,24,25]. The combination of both perspectives can thus be seen as a strength of our study.

Another strength is that households included in the study live in different informal settlements spread over both rural and urban areas and, overall, the nine provinces of South Africa. This most likely has led to study results that can be generalized to the rest of the South African population living in informal settlements.

A limitation of the study is the large number of missing values. Of the households, 59.7% had a missing value on at least one essential variable, and 28.7% had missing values on more than three of the essential variables. We decided to exclude households with more than three missing values and to impute missing data in case of one, two, or three missing values. Nevertheless, there is a possibility that the exclusion of households with more than three missing values has introduced a certain extent of selection bias.

Another limitation of the study is that information on income was only available at the household level, and due to the lack of information on the number of working household members, we did not include this variable in the analysis.

Finally, this study calculated concentration indices, which were not decomposed by population subgroups using statistical software, while this would have made it possible to determine the relative importance of factor components, such as the gender, age, and education level of the household head. For future studies, we would recommend the conduction of a complementary decomposition analysis.

## 5. Conclusions

Our study showed that the use of a piped tap on property was disproportionately concentrated among households with higher SES, while households with lower SES were often limited to the use of other inferior (less safe and distant) sources of drinking water. Furthermore, the use of inferior sources of drinking water was significantly associated with the age, marital status, education status, and employment status of the household head. Our results thus demonstrate the existence of economic, demographic, and social inequalities in the use of drinking water among inhabitants of informal settlements in South Africa.

In order to reduce those inequalities, it is necessary to install new tap water points in the informal settlements and thereby achieve a more equitable distribution of water points among households with higher and lower SES. The group with no access to piped tap water should be prioritized in those interventions because it faces major health threats due to water contamination. It is, however, essential to assure not only quality but also the proximity of the water sources. Thus, subsequent interventions should ensure access to piped tap water at least 200 m from the dwelling. Besides, educational interventions to create awareness about the potential health risks of using unsafe drinking water are needed. This could encourage households to make extra efforts to use safe water sources. To be effective, the above interventions should be part of integrated programs aiming to improve not only access to safe drinking water but also sanitation infrastructure and hygiene conditions, not only in informal settlements but also in other living areas affected by such problems.

Subsequent studies are needed to better understand the socio-economic inequalities in access to safe drinking water in informal settlements since data are currently lacking. We were, therefore, unable to establish the convergent validity of some of our findings, like those related to employment, marital status, age, and gender. Subsequent studies are also needed to monitor the use of drinking water sources and to provide evidence for future policy interventions.

## Figures and Tables

**Table 1:** Household characteristics and the distribution of type of drinking water use over the total group and over different population subgroups.

Household Characteristics	Total	Distribution of Type of Drinking Water Use			
		Piped Tap on Property (N = 739)	Nearby Public Tap (N = 810)	Distant Public Tap (N = 308)	No-Tap Water (N = 163)
TOTAL		36.0%	40.5%	15.3%	8.3%
HOUSEHOLD SIZE (N = 2284)					
<4 MEMBERS	50.1%	33.0%	42.2%	16.1%	8.7%
≥4 MEMBERS	49.9%	39.7%	38.5%	14.4%	7.4%
GENDER HH (N = 2167)					
MALE	52.1%	33.2%	41.0%	17.6%	8.1%
FEMALE	47.9%	38.9%	39.3%	13.7%	8.1%
AGE HH (N = 2163)					
<35 YEARS	31.9%	31.0%	44.6%	16.6%	7.8%
35–50 YEARS	39.0%	33.5%	42.7%	16.1%	7.7%
>50 YEARS	29.1%	45.5%	33.7%	12.6%	8.2%
NATIONALITY HH (N = 2243)					
SOUTH-AFRICAN	95.9%	36.7%	40.0%	15.4%	7.8%
OTHER	4.1%	28.4%	46.9%	9.9%	14.8%
MARITAL STATUS HH (N = 2243)					
PARTNER	48.1%	34.4%	41.7%	16.1%	7.9%
NO PARTNER	51.9%	38.7%	38.6%	14.5%	8.3%
EDUCATION STATUS HH (N = 2130)					
LOW	18.4%	36.0%	37.8%	13.8%	12.4%
INTERMEDIATE	39.9%	36.4%	40.7%	14.7%	8.2%
HIGH	41.7%	35.7%	42.6%	15.8%	5.9%
EMPLOYMENT STATUS HH (N = 2235)					
EMPLOYED	38.9%	31.5%	44.6%	15.8%	8.1%
NOT EMPLOYED	61.1%	38.8%	37.9%	15.2%	8.1%

The sample sizes with complete cases are presented; HH = household head.

**Table 2:** Results of multivariate multinomial logistic regression analyses showing the associations between household characteristics and drinking water use.

Household Characteristics	Piped Tap on Property as Reference Category					
	Nearby Public Tap		Distant Public Tap		No-Tap Water	
	OR (95%-CI)	*p*-Value	OR (95%-CI)	*p*-Value	OR (95%-CI)	*p*-Value
SOCIO-ECONOMIC STATUS	Reference category: fifth (highest SES)					
FIRST QUINTILE	5.12 (3.68, 7.12)	0.00 *	3.76 (2.53, 5.58)	0.00 *	8.91 (4.88, 16.28)	0.00 *
SECOND QUINTILE	4.06 (2.67, 6.19)	0.00 *	2.07 (1.19, 3.62)	0.01 *	5.66 (2.76, 11.60)	0.00 *
THIRD QUINTILE	1.99 (1.44, 2.76)	0.00 *	1.71 (1.12, 2.60)	0.01 *	2.44 (1.26, 4.73)	0.01 *
FOURTH QUINTILE	1.31 (0.96, 1.78)	0.09	0.90 (0.58, 1.40)	0.65	1.43 (0.72, 2.84)	0.31
HOUSEHOLD SIZE	Reference category: ≥4 members					
<4 MEMBERS	1.10 (0.88, 1.37)	0.42	1.14 (0.84, 1.53)	0.41	1.03 (0.72, 1.48)	0.86
GENDER HH	Reference category: female					
MALE	0.94 (0.74, 1.19)	0.60	1.22 (0.90, 1.66)	0.20	0.96 (0.65, 1.43)	0.86
AGE HH	Reference category: >50 years					
<35 YEARS	1.76 (1.29, 2.40)	0.00 *	1.76 (1.13, 2.75)	0.02 *	1.67 (0.99, 2.80)	0.06
35–50 YEARS	1.71 (1.28, 2.27)	0.00 *	1.73 (1.18, 2.56)	0.01 *	1.58 (1.00, 2.49)	0.05
NATIONALITY HH	Reference category: other					
SOUTH-AFRICAN	0.82 (0.48, 1.39)	0.46	1.49 (0.64, 3.45)	0.36	0.53 (0.25, 1.11)	0.09
MARITAL STATUS HH	Reference category: partner					
NO PARTNER	0.74 (0.58, 0.93)	0.01 *	0.76 (0.56, 1.02)	0.07	0.79 (0.55, 1.14)	0.21
EDUCATION STATUS HH	Reference category: high					
LOW	1.18 (0.82, 1.68)	0.37	1.18 (0.76, 1.84)	0.46	2.44 (1.40, 4.25)	0.00 *
INTERMEDIATE	1.04 (0.81, 1.34)	0.75	1.08 (0.77, 1.50)	0.66	1.39 (0.90, 2.15)	0.14
EMPLOYMENT STATUS HH	Reference category: employed					
UNEMPLOYED	0.68 (0.55, 0.85)	0.00 *	0.82 (0.61, 1.10)	0.19	0.71 (0.49, 1.02)	0.06
NAGELKERKE PSEUDO R^2^	0.14					

Results are presented as odds ratios (OR) and 95% confidence interval (95%-CI), with the use of a piped tap on property as the reference category. The model’s goodness-of-fit is calculated as the average Nagelkerke pseudo R^2^ of ten imputed datasets. * *p* < 0.05; HH = household head.

**Figure A1:**
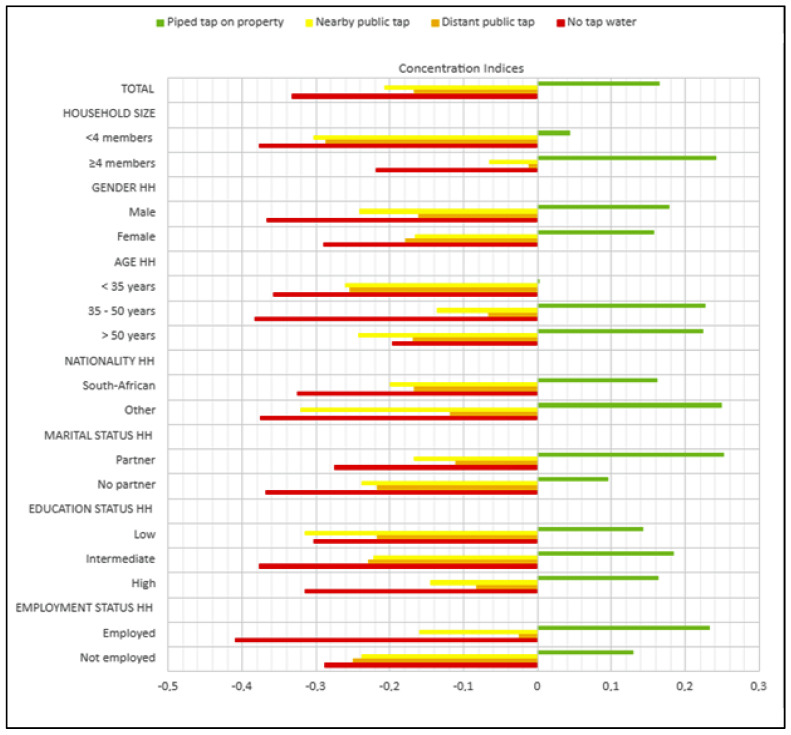
Concentration indices for different population subgroups; HH = household head.

## Data Availability

Information about data availability can be obtained from the authors.

## Appendix A. Concentration Indices for Different Population Subgroups

Results are presented as concentration indices. A positive value indicates that the use of that particular type of drinking water is disproportionately concentrated among households with higher socio-economic status, and a negative value that the use of that type of drinking water is disproportionately concentrated among people with lower socio-economic status.

## Appendix B. Univariate Multinomial Logistic Regression Analyses

**Table A1:** Results of univariate multinomial logistic regression analyses showing the associations between household characteristics and drinking water use.

Household Characteristics	Piped Tap on Property as Reference Category					
	Nearby Public Tap		Distant Public Tap		No Tap Water	
	OR (95%-CI)	*p*-Value	OR (95%-CI)	*p*-Value	OR (95%-CI)	*p*-Value
SOCIO-ECONOMIC STATUS	Reference category: fifth (highest SES)					
FIRST QUINTILE	5.00 (3.65, 6.86)	0.00 **	3.78 (2.58, 5.55)	0.00 **	9.19 (5.09, 16.60)	0.00 **
SECOND QUINTILE	4.05 (2.69, 6.10)	0.00 **	2.10 (1.21, 3.66)	0.01 **	5.74 (2.81, 11.70)	0.00 **
THIRD QUINTILE	1.91 (1.38, 2.63)	0.00 **	1.69 (1.12, 2.55)	0.01 **	2.42 (1.26, 4.67)	0.01 **
FOURTH QUINTILE	1.31 (0.96, 1.78)	0.09	0.90 (0.58, 1.40)	0.64	1.47 (0.74, 2.90)	0.27
GOODNESS-OF-FIT	0.11					
HOUSEHOLD SIZE	Reference category: ≥4 members					
<4 MEMBERS	1.33 (1.09, 1.61)	0.00 **	1.35 (1.04, 1.76)	0.03 **	1.39 (1.00, 1.93)	0.05 **
GOODNESS-OF-FIT	0.01					
GENDER HH	Reference category: female					
MALE	1.19 (0.98, 1.46)	0.09 *	1.49 (1.13, 1.96)	0.01 **	1.15 (0.81, 1.62)	0.45
GOODNESS-OF-FIT	0.01					
AGE HH	Reference category: >50 years					
<35 YEARS	1.92 (1.49, 2.48)	0.00 **	1.86 (1.27, 2.71)	0.00 **	1.38 (0.89, 2.14)	0.15
35–50 YEARS	1.72 (1.34, 2.19)	0.00 **	1.66 (1.17, 2.35)	0.01 **	1.26 (0.85, 1.88)	0.25
GOODNESS-OF-FIT	0.02					
NATIONALITY HH	Reference category: other					
SOUTH-AFRICAN	0.73 (0.44, 1.19)	0.21	1.30 (0.57, 2.95)	0.53	0.44 (0.22, 0.87)	0.02 **
GOODNESS-OF-FIT	0.01					
MARITAL STATUS HH	Reference category: partner					
NO PARTNER	0.83 (0.68, 1.01)	0.06 *	0.80 (0.62, 1.03)	0.08 *	0.92 (0.67, 1.28)	0.63
GOODNESS-OF-FIT	0.01					
EDUCATION STATUS HH	Reference category: high					
LOW	0.87 (0.65, 1.16)	0.34	0.86 (0.59, 1.26)	0.45	1.97 (1.26, 3.10)	0.00 **
INTERMEDIATE	0.92 (0.74, 1.16)	0.49	0.92 (0.68, 1.26)	0.61	1.31 (0.88, 1.94)	0.18
GOODNESS-OF-FIT	0.01					
EMPLOYMENT STATUS HH	Reference category: employed					
NOT EMPLOYED	0.68 (0.56, 0.84)	0.00 **	0.78 (0.59, 1.03)	0.08 *	0.81 (0.58, 1.14)	0.23
GOODNESS-OF-FIT	0.01					

Results are presented as odds ratios (OR) and 95% confidence interval (95%-CI), with the use of piped tap water on property as the reference category. The model’s goodness-of-fit was calculated as the average Nagelkerke pseudo R^2^ of ten imputed datasets. * *p* < 0.10 ** *p* < 0.05; HH = household head.
